# Supplementary material for: NOD2 deficiency increases retrograde transport of secretory IgA complexes in Crohn’s disease
Source: Nat Commun. 2021 Jan 11;12:261. doi: 10.1038/s41467-020-20348-0 (PMC7801705; doi:10.1038/s41467-020-20348-0)
Supplement: Supplementary file 1 — Supplementary Information [file 41467_2020_20348_MOESM1_ESM.pdf]

Supplemental Information

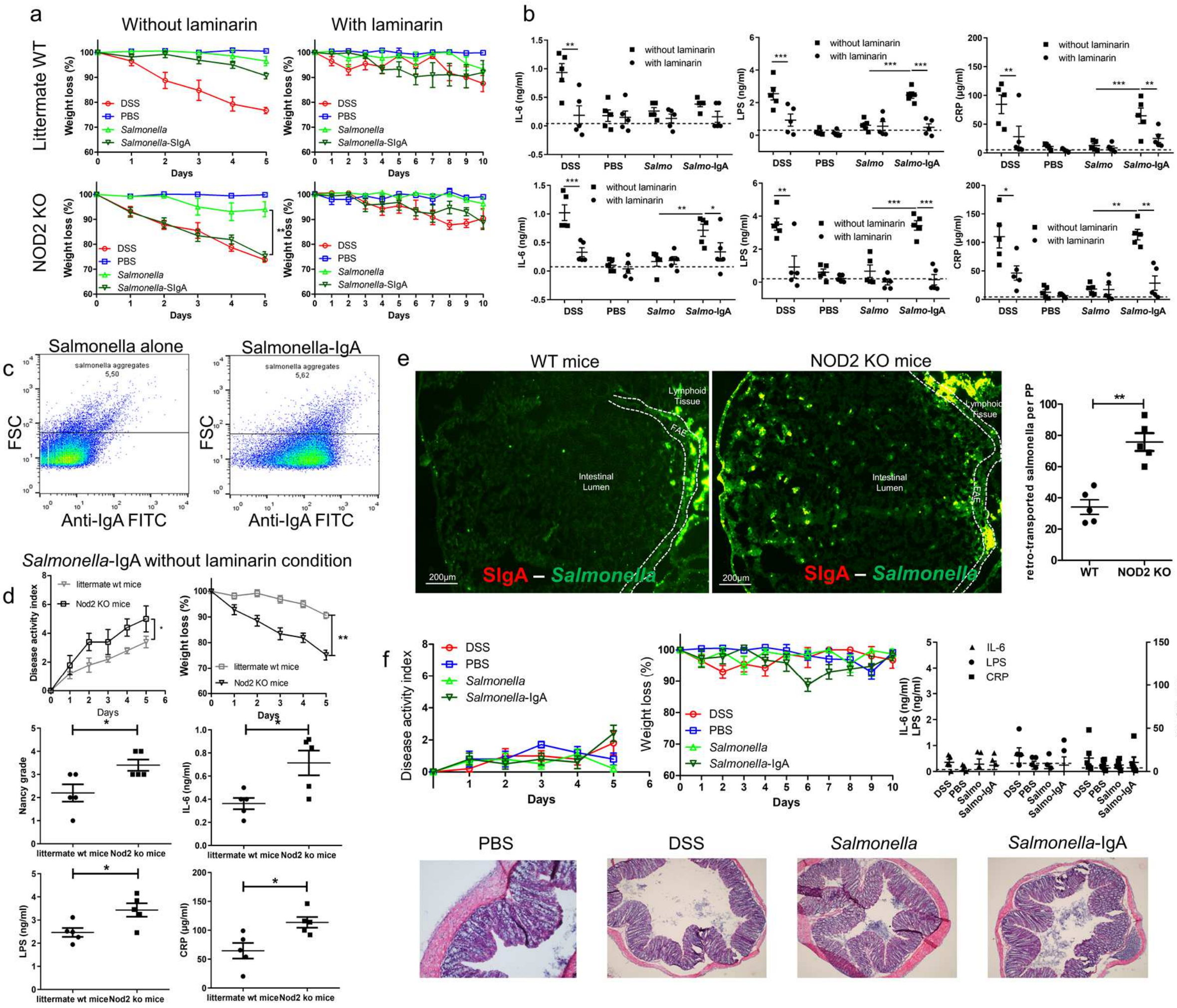

Supplementary Figure 1: Complementary results from the induction of colitis in mice. The clinical progression of colitis was also evaluated by comparing weight loss to initial weight (a) and by quantifying proinflammatory IL-6, LPS and CRP in serum (b). Vertical bars show the mean value +/- SEM. n=5 biologically independent mice. One-way ANOVA followed by Bonferroni post hoc test was used (*p* values: ** $p < 0.001$). (c) Using the same *in vitro* binding conditions, salmonella aggregates with or without IgASal4 were measured by flow cytometry. (d) Mice receiving Salmonella-IgA without laminarin were isolated from Fig 3a/c and supplemental Fig 1a/b and replotted in order to statistically analysed littermate WT and Nod2KO mice. n=5 biologically independent mice. Vertical bars show the mean value +/- SEM. A nonparametric Mann-Whitney U-test was used (*p* values: * $p < 0.05$; ** $p < 0.001$). (e) Tissue section showing a PP obtained from a ligated intestinal loop WT (left panel) or *NOD2* KO (right panel) following exposure SIgA-*Salmonella*(GFP) for 60 min. Tissue section were labeled with PE-anti-IgA at room temperature for 2 h. This experiment was repeated on 5 mice per group. Graph shows the retrotransported *Salmonella* per PP. n=5 biologically independent mice. Vertical bars show the mean value +/- SEM. A nonparametric Mann-Whitney U-test was used (*p* values: * $p < 0.05$; ** $p < 0.001$). (f) DAI, weight loss, serum IL-6, CRP, LPS, and neutrophil infiltrates (this experiment was repeated on 5 mice per group) in Dectin-1 KO mice after being orally challenged either with DSS, PBS, *Salmonella* Typhimurium or *Salmonella* Typhimurium bound to murine IgA. Vertical bars show the mean value +/- SEM. n=5 biologically independent mice. One-way ANOVA followed by Bonferroni post hoc test was used. Dotted lines represent the limit of ELISA detection.

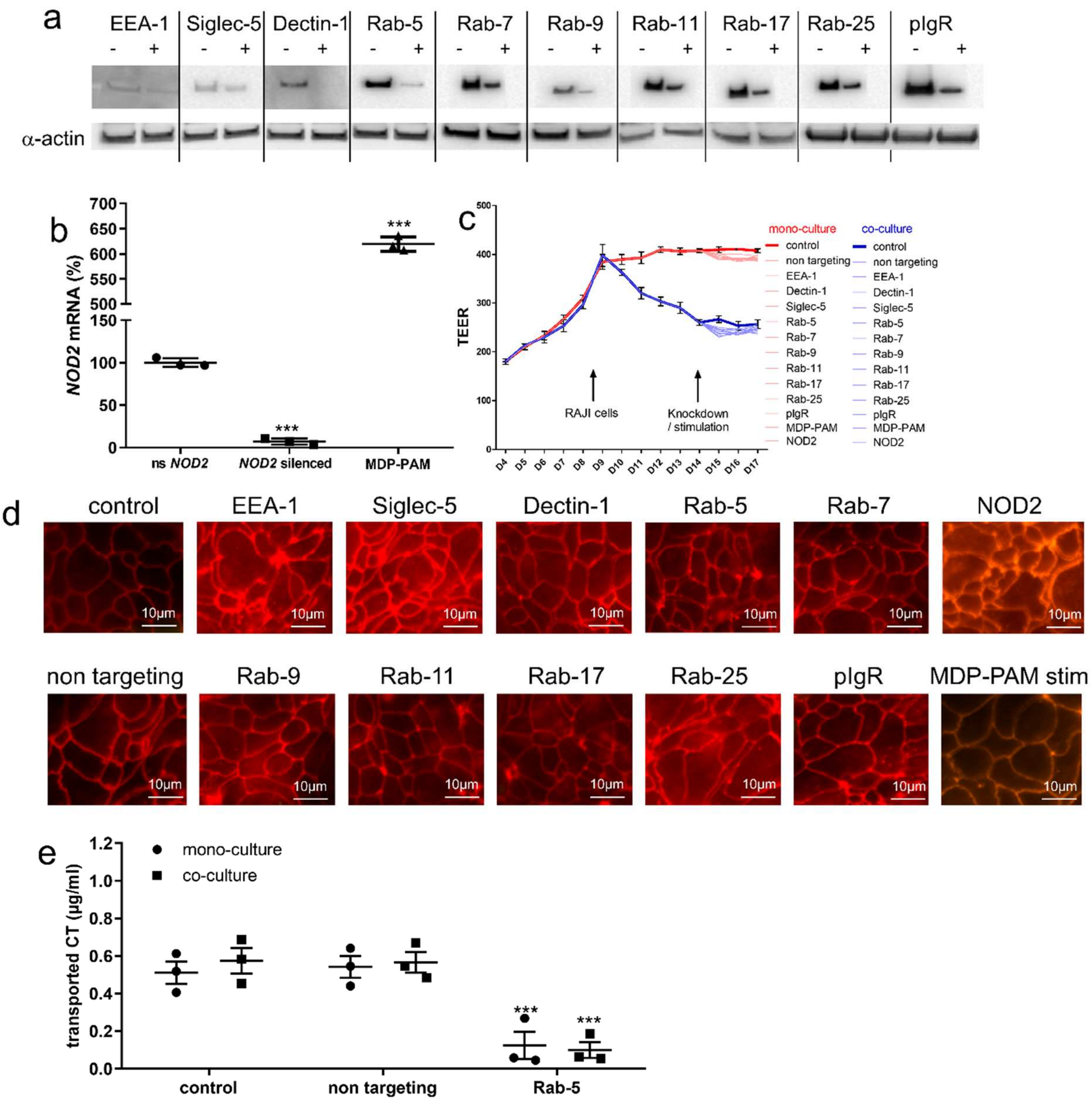

Supplementary figure 2: Confirmation of the effect of siRNA transfection in the inverted *in vitro* model of human FAE. (a) Western Blot show decrease of the amount of proteins between control "-" (no target knockdown) and knocked down cells "+". (b) Q-PCR shows decrease of the *NOD2* mRNA quantity between control and transfected cells and increase after 24 h stimulation with MDP-PAM. n=3 independent experiments. Vertical bars show the mean value ± SEM. A nonparametric Mann-Whitney U-test was used (*p* values: *** $p < 0.005$). (c) TEER is measured to detect the monolayer integrity and M cell conversion. No statistical changes between control and knockdown cells have been observed. n=3 independent experiments. A nonparametric Mann-Whitney U-test was used. (d) Tight junction staining confirms the monolayer integrity two days after siRNA transfection. This experiment was repeated twice with similar results. (e) After Rab-5 siRNA knockdown, CT transport was quantified in the inverted *in vitro* model of FAE. n=3 independent experiments. Vertical bars show the mean value ± SEM. One-way ANOVA followed by Bonferroni post hoc test was used (*** $p < 0.005$).
