# Supplementary material for: NOD2 deficiency increases retrograde transport of secretory IgA complexes in Crohn’s disease
Source: Nat Commun. 2021 Jan 11;12:261. doi: 10.1038/s41467-020-20348-0 (PMC7801705; doi:10.1038/s41467-020-20348-0)
Supplement: Supplementary file 3 — Reporting Summary [file 41467_2020_20348_MOESM3_ESM.pdf]

nature research

Corresponding author(s): Nicolas Rochereau, Xavier Roblin, Eva Michaud, Rémi Gayet, Blandine Chanut, Fabienne Jospin, Blaise Corthésy & Stéphane Paul

Last updated by author(s): 2020/03/22

Reporting Summary

Nature Research wishes to improve the reproducibility of the work that we publish. This form provides structure for consistency and transparency in reporting. For further information on Nature Research policies, see Authors & Referees and the Editorial Policy Checklist.

Statistics

For all statistical analyses, confirm that the following items are present in the figure legend, table legend, main text, or Methods section.

n/a	Confirmed	
☐	☒	The exact sample size (*n*) for each experimental group/condition, given as a discrete number and unit of measurement
☐	☒	A statement on whether measurements were taken from distinct samples or whether the same sample was measured repeatedly
☐	☒	The statistical test(s) used AND whether they are one- or two-sided<br>*Only common tests should be described solely by name; describe more complex techniques in the Methods section.*
☐	☒	A description of all covariates tested
☐	☒	A description of any assumptions or corrections, such as tests of normality and adjustment for multiple comparisons
☐	☒	A full description of the statistical parameters including central tendency (e.g. means) or other basic estimates (e.g. regression coefficient) AND variation (e.g. standard deviation) or associated estimates of uncertainty (e.g. confidence intervals)
☐	☒	For null hypothesis testing, the test statistic (e.g. *F*, *t*, *r*) with confidence intervals, effect sizes, degrees of freedom and *P* value noted<br>*Give P values as exact values whenever suitable.*
☐	☒	For Bayesian analysis, information on the choice of priors and Markov chain Monte Carlo settings
☐	☒	For hierarchical and complex designs, identification of the appropriate level for tests and full reporting of outcomes
☐	☒	Estimates of effect sizes (e.g. Cohen's *d*, Pearson's *r*), indicating how they were calculated

Our web collection on statistics for biologists contains articles on many of the points above.

Software and code

Policy information about availability of computer code

Data collection	BD FACSDiva™ Software
Data analysis	FlowJo™ v10.6.2, image J 1.46R

For manuscripts utilizing custom algorithms or software that are central to the research but not yet described in published literature, software must be made available to editors/reviewers. We strongly encourage code deposition in a community repository (e.g. GitHub). See the Nature Research guidelines for submitting code & software for further information.

Data

Policy information about availability of data

All manuscripts must include a data availability statement. This statement should provide the following information, where applicable:

- Accession codes, unique identifiers, or web links for publicly available datasets
- A list of figures that have associated raw data
- A description of any restrictions on data availability

The authors declare that the data supporting the findings of this study are available within the paper [and its supplementary information files]

Field-specific reporting

Please select the one below that is the best fit for your research. If you are not sure, read the appropriate sections before making your selection.

☒ Life sciences ☐ Behavioural & social sciences ☐ Ecological, evolutionary & environmental sciences

Life sciences study design

All studies must disclose on these points even when the disclosure is negative.

Sample size	sample size was chosen to ensure adequate power. Moreover, it's a pathophysiological proof-of-concept study. The number of patients was chosen based on a statistical hypothesis described in the manuscript.
Data exclusions	no data were excluded from the analyses
Replication	the number of technical and experimental replicas are indicated in the manuscript. Moreover, all attemps at replication were successful.
Randomization	all the mice were randomly assigned to the groups. N/A for the human study
Blinding	For human study, Nancy histological score has been evaluated by a blinded pathologist. For mice experiments, weights, biological measurements were performed blindly. All immunofluorescence analysis were also performed blindly.

Reporting for specific materials, systems and methods

We require information from authors about some types of materials, experimental systems and methods used in many studies. Here, indicate whether each material, system or method listed is relevant to your study. If you are not sure if a list item applies to your research, read the appropriate section before selecting a response.

Materials & experimental systems

n/a	Involved in the study	
☐	☒	Antibodies
☐	☒	Eukaryotic cell lines
☒	☐	Palaeontology
☐	☒	Animals and other organisms
☐	☒	Human research participants
☒	☐	Clinical data

Methods

n/a	Involved in the study	
☒	☐	ChIP-seq
☐	☒	Flow cytometry
☒	☐	MRI-based neuroimaging

Antibodies

Antibodies used	anti-human PE-GP2 mAb (MBL, catalog D277-5, clone 3G7-H9),<br>anti-human PE-DC-SIGN mAb (ThermoFisher scientific, Catalog # 12-5888-42, clone MIH18),<br>Goat anti-human Dectin-1/CLEC 7A (R&D System, catalog AF1859)<br>anti-human CD170 (Siglec-5) mAb (mouse IgG1, R&D System, catalog MAB1072, Clone #194128)<br>sheep anti-human EEA-1 (R&D System, catalog AF8047)<br>anti-human pIgR mAb (Mouse IgG3, R&D System, catalog MAB2717, Clone # 825724)<br>anti-human SYK mAb (mouse IgG1, R&D System, catalog MAB7166, Clone # 720402)<br>anti-human TAK1 mAb (mouse IgG1, R&D System, catalog MAB5307, Clone # 491840)<br>rabbit anti-human Rab-5 (Abcam, catalog ab18211)<br>anti-human Rab-7 mAb (mouse IgG2b, Abcam, catalog ab50533, Clone # Rab-7-117)<br>anti-human Rab-9 mAb (mouse IgG1, Abcam, Catalog ab2810, clone # Mab9)<br>rabbit anti-human Rab-11 (Abcam, catalog ab3612)<br>anti-human Rab-25 mAb (Rabbit IgG, Abcam, catalog ab218624, Clone EPR18353)<br>GFP-IgA2 (Invivogen, catalog htnfa-mab7)
Validation	each antibody was used according to the manufacturer<br>anti-human PE-GP2 mAb (https://www.mblbio.com/bio/g/dtl/A/index.html?pcd=D277-5)<br>anti-human PE-DC-SIGN mAb (https://www.thermofisher.com/antibody/product/CD273-B7-DC-Antibody-clone-MIH18-Monoclonal/12-5888-42)<br>Goat anti-human Dectin-1/CLEC 7A (https://www.rndsystems.com/products/human-dectin-1-clec7a-antibody_af1859)<br>anti-human CD170 (Siglec-5) mAb (https://www.rndsystems.com/products/human-siglec-5-cd170-antibody-194117_mab1072)<br>sheep anti-human EEA-1 (https://www.rndsystems.com/products/human-mouse-rat-eea1-antibody_af8047)<br>anti-human pIgR mAb (https://www.rndsystems.com/products/human-pigr-antibody-825724_mab2717)<br>anti-human SYK mAb (https://www.rndsystems.com/products/human-syk-sh2d2-domain-antibody-720402_mab7166)<br>anti-human TAK1 mAb (https://www.rndsystems.com/products/human-tak1-antibody-491840_mab5307)<br>rabbit anti-human Rab-5 (https://www.abcam.com/rab5-antibody-early-endosome-marker-ab18211.html)<br>anti-human Rab-9 mAb (https://www.abcam.com/rab9-antibody-mab9-ab2810.html)<br>rabbit anti-human Rab-11 (https://www.abcam.com/rab11-antibody-ab3612.html)<br>anti-human Rab-25 mAb (https://www.abcam.com/rab5-antibody-epr21801-ab218624.html)<br>GFP-IgA2 (https://www.invivogen.com/anti-htnfa-higa2).

Eukaryotic cell lines

Policy information about cell lines

Cell line source(s)	human intestinal cell line Caco-2 cell (clone 1) (obtained from Dr. Maria Rescigno, University of Milan-Bicocca, Milan, Italy)<br>The human Burkitt's lymphoma cell line Raji B (American Type Culture Collection)
Authentication	none of the cell line were authenticated
Mycoplasma contamination	all cell lines were negative for mycoplasma contamination.
Commonly misidentified lines<br>(See ICLAC register)	no commonly misidentified cell lines were used in the study.

Animals and other organisms

Policy information about studies involving animals; ARRIVE guidelines recommended for reporting animal research

Laboratory animals	NOD2 KO mice were obtained from Gabriel Nunez (University of Michigan, USA). Dectin-1 KO mice were obtained from Gordon D. Brown (University of Aberdeen, UK). Littermate mice were obtained from Nod2-heterozygous crosses. All mice (8 weeks old, female) were hosted at the University Hospital Unit for animal testing (Saint-Etienne, France) in the same conditions.
Wild animals	no wild animals were used in the study
Field-collected samples	no field collected samples were used in the study.
Ethics oversight	The experimental procedures followed the protocol of the Ethics Committee of CREEA (Permit Number No. 69387487).

Note that full information on the approval of the study protocol must also be provided in the manuscript.

Human research participants

Policy information about studies involving human research participants

Population characteristics	the population characteristics were either Crohn's Disease patients or healthy individuals : age between 50 to 75 years old, either male or female without any specific genotypic information.
Recruitment	Informed and consenting CD patients or healthy individuals who had undergone lower endoscopy for routine diagnostic purposes with normal intestinal mucosa provided two biopsy samples from the terminal ileum.
Ethics oversight	Centre National Informatique et Liberte CNIL (Number: 1849323)<br>Ethic comitee of the CHU Saint-Etienne, Terre d'Ethique

Note that full information on the approval of the study protocol must also be provided in the manuscript.

Flow Cytometry

Plots

Confirm that:

☒ The axis labels state the marker and fluorochrome used (e.g. CD4-FITC).

☒ The axis scales are clearly visible. Include numbers along axes only for bottom left plot of group (a 'group' is an analysis of identical markers).

☒ All plots are contour plots with outliers or pseudocolor plots.

☒ A numerical value for number of cells or percentage (with statistics) is provided.

Methodology

Sample preparation	Fecal supernatant from WT and Nod2KO mice were washed, filtered and stained with anti-IgA-FITC
Instrument	FACS calibur
Software	Diva
Cell population abundance	no sort was realized before flow cytoletry analysis. bacteria contained in mice feces were in excess.

Gating strategy

IgA-bacteria complexes were visualized using FSC and anti-IgA FITC. boundaries between positive and negative staining cell populations is defined in the figure.

☒ Tick this box to confirm that a figure exemplifying the gating strategy is provided in the Supplementary Information.
